# Simple and inexpensive synovial fluid biomarkers for the diagnosis of prosthetic joint infection according to the new EBJIS definition

**DOI:** 10.5194/jbji-8-109-2023

**Published:** 2023-03-29

**Authors:** Sara Elisa Diniz, Ana Ribau, André Vinha, José Carlos Oliveira, Miguel Araújo Abreu, Ricardo Sousa

**Affiliations:** 1 Orthopedics Department, Centro Hospitalar Universitário de Santo António, Porto, Portugal; 2 Department of Infectious Diseases, Centro Hospitalar Universitário de Santo António, Porto, Portugal; 3 Department of Laboratory Pathology, Centro Hospitalar Universitário de Santo António, Porto, Portugal; ℹ part of the Porto Bone and Joint Infection Group (GRIP), Porto, Portugal

## Abstract

**Introduction**: diagnosis of periprosthetic joint infection (PJI) is challenging, as no single test has absolute accuracy. The purpose of this study was to assess the utility of different simple synovial biomarkers in the diagnosis of PJI as defined by the European Bone and Joint Infection Society (EBJIS). **Methods**: we retrospectively identified all patients undergoing revision hip or knee arthroplasty from 2013 to 2019 on our prospectively maintained database. Only patients with minimum required infection diagnostic workup were included in the study. Patients with comorbidities that may influence the accuracy of synovial biomarkers were excluded. Receiver operator characteristic (ROC) curves were utilised to assess the diagnostic utility of synovial fluid white blood cell (WBC) count, polymorphonuclear leukocyte percentage (PMN %), C-reactive protein (CRP), adenosine deaminase (ADA), and alpha-2-microglobulin (A2M). **Results**: in total, 102 patients met the inclusion criteria. Of these, 58 were classified as infection unlikely, 8 as infection likely, and 36 as infection confirmed. Synovial WBC count (area under the curve (AUC) 0.94) demonstrated the best utility for the diagnosis of PJI, followed by PMN % (AUC 0.91), synovial CRP (AUC 0.90), ADA (AUC 0.82), and A2M (AUC 0.76). We found added value in the combined interpretation of different biomarkers. We calculated high sensitivity and negative predictive value if at least two of them are negative and high specificity and positive predictive value if at least two are elevated. **Conclusion**: current results show that synovial fluid investigation is a useful tool for the diagnosis of PJI, and the combined interpretation of simple and inexpensive biomarkers demonstrated improved diagnostic accuracy.

## 1 Introduction

Total joint arthroplasty (TJA) is among the most successful procedures in orthopaedics, and demand for this surgery is expected to continue to rise worldwide (Rupp et al., 2020; Singh et al., 2019). Revision arthroplasty is subsequently, also on the rise. Periprosthetic joint infection (PJI) is a devastating complication and a leading cause of failure following TJA. It is frequently present even in presumed aseptic cases (Hipfl et al., 2021; Jacobs et al., 2017). It is now recognised that making an accurate diagnosis of PJI is paramount to ensuring treatment success.

Despite advancements in technology, a “gold standard” test with perfect diagnostic accuracy has not been identified to date. Hence, physicians often rely on a combination of clinical, laboratory, and intraoperative findings. Notwithstanding, the diagnosis of PJI is often challenging, and it can easily be missed if a high index of suspicion is not adopted (Hipfl et al., 2021; Jacobs et al., 2017).

Recently, the European Bone and Joint Infection Society (EBJIS) proposed a three-level diagnostic approach based on classic clinical, laboratory, and radiographic findings (McNally et al., 2021). This definition divides cases into unlikely infection, confirmed infection, and proposes a novel third group as likely but not confirmed PJI. The EBJIS definition has been suggested to have increased sensitivity compared to previously proposed criteria, without negatively impacting specificity. It has also been showed to be better in preoperatively ruling out PJI when serological and synovial biomarkers yield negative results (Sigmund et al., 2022; Sousa et al., 2023).

However, in cases of preoperative likely but not confirmed infection, a doubt arises about how to proceed. To help decision making in this group, it is possible to use alternative biomarkers in addition to differential leukocyte count. Alpha-defensin is such an example but has important limitations. It is not only too expensive to merit routine use (Kleeman-Forsthuber et al., 2021), but it has also been shown to have limited sensitivity if you use more sensitive definitions such as the EBJIS (Renz et al., 2018). We have previously shown that adding simple, inexpensive biomarkers that we can easily measure in our laboratory, such as synovial fluid C-reactive protein (CRP), adenosine deaminase (ADA), and alpha-2-macrogloblulin (A2M), does contribute to more accurate diagnosis (Sousa et al., 2017).

Our hypothesis for this study is that using these same biomarkers can be helpful when using the EBJIS PJI definition, especially if you get an intermediate differential leukocyte result. As such, our goal was to revise the role of combined biomarker interpretation in diagnosing PJI as defined by the EBJIS criteria.

## 2 Patients and methods

We performed a retrospective review of our institution's prospective database. We included all patients that underwent total hip or knee arthroplasty revision surgery (regardless of preoperative diagnosis) at our institution between January 2013 and December 2019. We received Institutional Review Board (IRB) approval prior to the initiation of the present study.

Data concerning patient demographics and original joint replacement surgery were collected. Detailed clinical information before revision surgery was exhaustively collected with a special emphasis on variables relevant for the diagnosis of PJI (e.g. presence of sinus tract, history of recent fever or bacteraemia, antibiotic therapy at the time of surgery, and blood inflammatory parameters). Synovial fluid investigation results, intraoperative findings (e.g. purulence), and definitive microbiologic and histological results were also recorded.

Cases without the minimum required diagnostics to classify them as aseptic are as follows: less than four intraoperative microbiology samples (synovial fluid, tissue samples, implant sonication) ( N=183 ) and no preoperative/intraoperative synovial fluid differential leukocyte count ( N=68 ) were excluded. To reduce bias, we also excluded cases with conditions that influence synovial fluid testing accuracy (i.e. inflammatory arthritis, metal-on-metal bearing, periprosthetic fracture, antibiotic within 2 weeks prior to revision surgery, revision surgery less than 6 weeks after index procedure, and acute hematogenous infections with less than 4 weeks of symptoms) ( N=11 ).

In addition to culture and differential leukocyte count, other synovial fluid biomarkers were routinely measured: C-reactive protein (CRP), adenosine deaminase (ADA), and alpha-2-macroglobulin (A2M). The laboratory methodology for these measurements was previously described (Sousa et al., 2017).

After application of the new EBJIS PJI definition, we categorised our patient population into three distinct groups: (1) unlikely infection, (2) likely infection, or (3) confirmed infection. A comparison of results was made between the three groups to calculate values of sensitivity and negative predictive values (NPV) and specificity and positive predictive values (PPV).

## 3 Statistical analysis

Categorical variables are expressed as counts (percentage), and frequency distributions were compared with the chi-squared test. Continuous variables were expressed as mean values (interquartile range). Analysis of variance (ANOVA) was used to analyse the differences among means. Optimal cutoff values were determined using receiver operating characteristic (ROC) curve analysis. These curves were generated, and the areas under the curve were compared to determine the most appropriate cutoff. Specificity and predictive values of the tests were estimated. Using selected cutoff values, multiple combinations were created, with the aim of improving the ability to confirm or exclude the diagnosis of PJI. These values were obtained by regrouping unlikely–likely group and confirmed group to get more sensitive values, and unlikely group and likely–confirmed group to get more specific values. The statistical tests used were two-tailed, and a p value <0.05 was considered statistically significant. The correlation coefficient was calculated using Cohen's kappa to obtain the agreement values between biomarkers.

**Figure 1:**
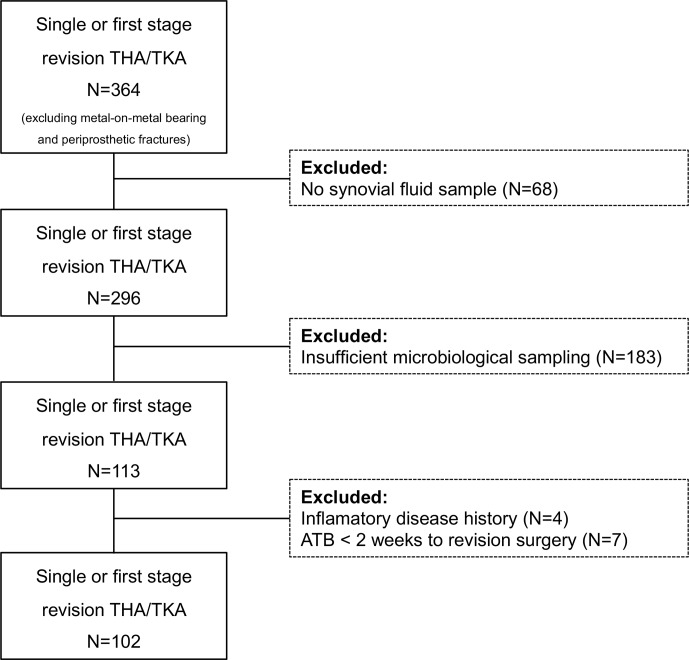
Flowchart of inclusion criteria.

**Table 1:** Demographic and clinical information of the patients ${}^{a}$ .

	Unlikely	Likely	Confirmed	p value
	n=58	n=8	n=36	
Age (years)	62.4 ( ±8.5 )	67.6 ( ±6.4 )	62.5 ( ±11.7 )	0.69 ${}^{b}$
Gender (female) (%)	52 (89.7)	8 (100)	24 (66.7)	<0.001 ${}^{c}$
Hip : knee ratio	5:53	1:7	14:32	0.028 ${}^{c}$
Primary : revision prosthesis ratio	54:6	9:2	37:9	0.359 ${}^{c}$
ERS serum (mm h ${}^{-1}$ )	24.9 ( ±18.6 )	40.2 ( ±33.3 )	54.7 ( ±30.4 )	<0.001 ${}^{b}$
	n=37	n=8	n=34	
CRP serum (mg L ${}^{-1}$ )	7.8 ( ±14,5 )	11.2 ( ±13.2 )	52.5 ( ±76.2 )	<0.001 ${}^{b}$
	n=38	n=8	n=36	

${}^{a}$ Expressed as mean ( ± standard deviation). ${}^{b}$ ANOVA test. ${}^{c}$ Chi-squared test, ERS – erythrocyte sedimentation rate, and CRP – C-reactive protein.

## 4 Results

Of the 364 revision arthroplasties we identified, only 102 met our inclusion criteria (Fig. 1). After applying the EBJIS definition, we classified 58 cases as infection unlikely, 8 as likely infections, and 36 confirmed infection cases.

Inflammatory serum markers were significantly different between groups, with an increasing trend between them. Other demographic and clinical information of the patients are shown in Table 1.

Synovial fluid investigation results are expressed in Table 2. All studied parameters were significantly increased in the confirmed infection group with an intermediate result in infection-likely cases when compared to unlikely infections.

**Figure 2:**
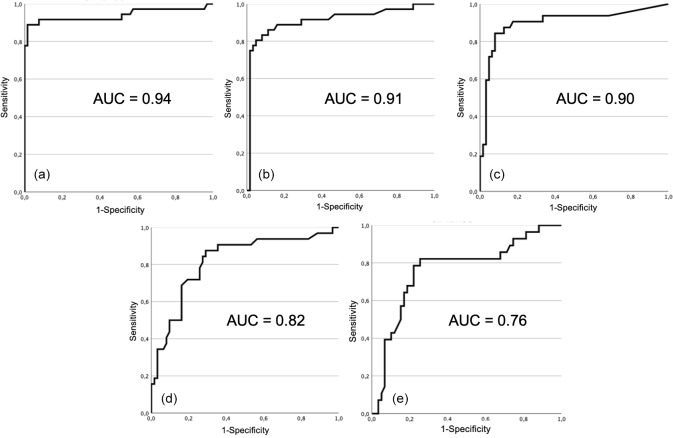
Receiver operating characteristic (ROC) curve and area under curve (AUC) for each test: **(a)** total leucocyte count, **(b)** proportion of PMN, **(c)** synovial fluid CRP, **(d)** adenosine deaminase, and **(e)** alpha-2-macroglobulin.

Receiver operating characteristic (ROC) curves for each test is shown in Fig. 2. Area under the curve was higher for total leukocyte count (0.94), proportion of PMN (0.91), synovial fluid CRP (0.90), followed by adenosine deaminase (0.82), and lastly alpha-2-macroglobulin (0.76). The optimal cutoffs found, as determined by each respective ROC curve, were 1470 cells µ L ${}^{-1}$ for total leucocyte count, 62.5 % for proportion of PMN, 2.7 mg L ${}^{-1}$ for CRP, 60 U L ${}^{-1}$ for ADA, and 420 mg L ${}^{-1}$ for A2M.

Table 3 shows the diagnostic performance of these cutoffs and the performance of different cutoffs selected by its resemblance to EBJIS-adopted cutoffs/philosophy. In general, lower cutoffs revealed high sensitivity and NPV (i.e. good rule-out accuracy), and higher interval cutoffs revealed high specificity and PPV (i.e. good rule-in accuracy).

The concordance coefficient (kappa) between different parameters was calculated to be 0.694 between total leucocyte count and PMN, 0.685 between total leucocyte count and CRP, and 0.616 between PMN and CRP. They show a strong though not perfect agreement between them, justifying their combined analysis to increase diagnostic yield.

As such, in line with our proposed goal, we combined parameters, considering associations where either one or other of the markers were positive to increase the sensitivity, or alternatively, where two or more markers were positive, to increase specificity.

Table 4 shows diagnostic accuracy using combinations of positive lower cutoffs (i.e. enough to classify cases as infection-likely but not confirmed). It is noteworthy that by combining classic parameters' lower cutoffs such as total leukocyte count or proportion of PMN with other positive lower cutoffs, we got high values of specificity and PPV for affirming PJI.

**Table 2:** Synovial fluid analysis results according to the presence of infection ${}^{a}$ .

EBJIS PJI classification	Unlikely	Likely	Confirmed	p value ${}^{b}$
	n=58	n=8	n=36	
Total leukocyte count (cells µ L ${}^{-1}$ )	427.0 ( ±408.5 )	1193.1 ( ±949.9 )	22 820.8 ( ±21354.7 )	<0.0001
	n=58	n=8	n=36	
Proportion of PMN (%)	26.4 ( ±23.2 )	52.4 ( ±25. )	81.5 ( ±24.3 )	<0.0001
	n=54	n=8	n=36	
C-reactive protein (mg L ${}^{-1}$ )	0.87 ( ±1.9 )	7.4 ( ±13.6 )	30.7 ( ±56.3 )	<0.0001
	n=54	n=9	n=32	
Adenosine deaminase (U L ${}^{-1}$ )	41.4 ( ±33.6 )	43.7 ( ±24.3 )	112.6 ( ±113.4 )	<0.0001
	n=54	n=8	n=32	
α -2-macroglobulin (mg L ${}^{-1}$ )	407.5 ( ±477.2 )	504.8 ( ±491.2 )	834.0 ( ±491.0 )	<0.0001
	n=51	n=8	n=28	

${}^{a}$ Expressed as mean ( ± standard deviation). ${}^{b}$ Kruskal–Wallis test. PMN – polymorphonuclear neutrophils.

**Table 3:** Statistically optimal and selected rule-in and rule-out cutoff diagnostic performance.

	Proposed cutoff(s)	Sensitivity	Specificity	Positive predictive	Negative predictive
				value	value
Total leukocyte count (cells µ L ${}^{-1}$ )	1470 ${}^{*}$	91.7 %	92.4 %	88.7 %	94.5 %
	2645	77.8 %	98.5 %	97.1 %	87.3 %
	3280	77.8 %	100 %	100 %	87.4 %
Proportion of PMN (%)	62.5 ${}^{*}$	88.9 %	83.9 %	78.1 %	92.1 %
	64.4	86.1 %	88.7 %	83.2 %	90.8 %
	79.5	75.0 %	98.4 %	96.8 %	85.9 %
C-reactive protein (mg L ${}^{-1}$ )	1.2	90.6 %	82.5 %	77.0 %	93.1 %
	2.7 ${}^{*}$	84.4 %	92.1 %	87.4 %	90.1 %
	8.1	71.9 %	95.2 %	90.7 %	83.9 %
Adenosine deaminase (U L ${}^{-1}$ )	40	90.6 %	64.5 %	62.3 %	91.4 %
	60 ${}^{*}$	71.9 %	80.6 %	70.6 %	81.6 %
	85	50.0 %	90.3 %	76.9 %	73.6 %
α -2-macroglobulin (mg L ${}^{-1}$ )	420 ${}^{*}$	82.1 %	74.6 %	67.7 %	86.6 %
	985	39.3 %	93.2 %	78.9 %	70.3 %

${}^{*}$ Optimal ROC curve proposed values, PMN – polymorphonuclear neutrophils, CRP – C-reactive protein, and ADA – adenosine deaminase.

**Table 4:** Diagnostic accuracy of selected test(s) values and respective combinations.

	Sensitivity	Specificity	Positive predictive	Negative redictive
	(95 % CI)	(95 % CI)	value (95 % CI)	value (95 % CI)
One of the test(s) is positive				
Leukocyte count >1470 or CRP >2.7 mg L ${}^{-1}$	97.3 %	86.8 %	80.0 %	98.3 %
	(85.8–99.9)	(76.4–93.9)	(68.5–88.1)	(89.5–99.8)
Leukocyte count >1470 or ADA >60	94.6 %	76.1 %	68.4 %	96.3 %
	(81.8–99.3)	(64.1–85.7)	(58.3–76.9)	(86.6–99.0)
PMN >62.5% or CRP >2.7 mg L ${}^{-1}$	89.5 %	79.4 %	70.3 %	93.3 %
	(75.2–97.1)	(67.9–88.3)	(59.5–79.3)	(84.5–97.2)
PMN >62.5% or ADA >60 U L ${}^{-1}$	92.1 %	69.1 %	61.9 %	94.1 %
	(78.6–98.3)	(56.7–79.8)	(53.0–70.2)	(84.3–98.0)
Both test(s) positive				
Leukocyte count >1470 and PMN >62.5%	85.3 %	96.7 %	93.4 %	92.3 %
	(68.9–95.0)	(88.6–99.6)	(78.3–98.2)	(84.3–96.4)
Leukocyte count >1470 and CRP >2.7 mg L ${}^{-1}$	77.4 %	98.3 %	96.1 %	88.9 %
	(58.9–90.4)	(90.9–100.0)	(78.0–99.4)	(80.6–93.9)
Leukocyte count >1470 and ADA >60 U L ${}^{-1}$	64.5 %	96.7 %	91.5 %	83.3 %
	(45.4–80.8)	(88.6–99.6)	(72.8–97.7)	(75.6–89.0)
PMN >62.5% and CRP >2.7 mg L ${}^{-1}$	83.3 %	98.3 %	96.4 %	93.0 %
	(65.3–94.4)	(90.8–100.0)	(79.0–99.5)	(85.5–97.4)
PMN >62.5% and ADA >60 U L ${}^{-1}$	66.7 %	98.2 %	95.3 %	84.4 %
	(47.2–82.7)	(90.4–100.0)	(74.2–99.3)	(76.5–90.0)
Three of the tests positive				
Leuc >1470 and PMN >62.5% and CRP >2.7	76.67 %	100 %	100 %	89.1 %
	(57.7–90.1)	(92.9–100.0)	(100.0)	(81.0–94.0)
Leuc >1470 and PMN >62.5% and ADA >60	60.7 %	100 %	100 %	82.5 %
	(40.6–78.5)	(92.5–100)	(100.0)	(75.0–88.2)

PMN – polymorphonuclear neutrophils, CRP – C-reactive protein, and ADA – adenosine deaminase.

## 5 Discussion

It is well-established that PJI can be present in a significant proportion of presumed aseptic cases (Hipfl et al., 2021; Jacobs et al., 2017; Portillo et al., 2013; Ribera et al., 2014). Accurate diagnosis is paramount, as inappropriate treatment may negatively impact outcomes (Staats et al., 2017; Milandt et al., 2019; Vargas-Reveron et al., 2020). Notwithstanding, due to the lack of a gold standard test, the diagnosis of PJI remains challenging.

Definitive diagnosis must therefore rely on a set of predetermined criteria that constitute any given definition and include intraoperative findings such as microbiological and histological results (McNally et al., 2021; Shohat et al., 2019; Osmon et al., 2013; Parvizi et al., 2011). Although there is no universally accepted algorithm for the diagnosis of PJI, it is well-established that arthrocentesis, and subsequent synovial fluid analysis, is essential in the workup of patients with suspected PJI.

The present study demonstrated that it is possible to reliably extract information to rule out and affirm infection from simple and inexpensive synovial fluid investigation, such as routine differential leukocyte count. Several different tests can be performed on synovial fluid to aid in the diagnosis of PJI. Traditional microbiological culture is still the gold standard for pathogen identification. However, it can take up to several days to produce a result. Furthermore, synovial fluid cultures often lack sensitivity, especially in cases of chronic low-grade infections caused by low-virulent microorganisms (Qu et al., 2013). Notwithstanding, it is still of major importance, especially in cases where no fluid can be gathered (so-called dry tap) and lavage–reaspiration is performed (Partridge et al., 2018; Li et al., 2019).

Due to its high diagnostic accuracy and widespread availability, synovial fluid total leukocyte and differential counts have become the cornerstone of PJI diagnosis in the last few years. However, there remains controversy surrounding the ideal cutoff for these tests. It has been suggested that cutoffs may vary by joint (hips or knees), infecting microorganism, and laboratory measurement protocols (Ottink et al., 2019). A number of different optimal cutoffs have therefore been proposed, ranging from 1100 to over 4200 cells µ L ${}^{-1}$ (Sousa et al., 2017; Ottink et al., 2019; Schinsky et al., 2008; Ghanem et al., 2008; Dinneen et al., 2013; De Vecchi et al., 2018; Zahar et al., 2018). The recent EBJIS PJI definition adopted a three-level distinction considering a low total leukocyte count <1500 cells µ L ${}^{-1}$ or proportion of PMN <65% to be sensitive enough to make infection unlikely (if there is no other positive feature present), and a high total leukocyte count >3000 cells µ L ${}^{-1}$ or proportion of PMN >80% to be specific enough to affirm infection (McNally et al., 2021).

The results of this paper seem to support the cutoffs advocated by the EBJIS definition. After extensive intraoperative investigation in every case that was included, the optimal total leukocyte count, in this case, 1470 cells µ L ${}^{-1}$ , demonstrated very high sensitivity and NPV. On the other hand, a cutoff closer to the traditional 3000 cells µ L ${}^{-1}$ threshold displayed very high specificity and PPV in the study population. Similar results were found with the proportion of PMN cutoff values. However, this results in a grey area (between 1500–3000 cells µ L ${}^{-1}$ ) where cell count interpretation is controversial.

Moreover, leukocyte count is subject to a number of significant limitations in cases of inflammatory arthritis and metallosis (Kwon et al., 2016; Qin et al., 2022). Thus, further driving the search for alternative biomarkers. Many different synovial fluid biomarkers are being investigated and a comprehensive discussion is beyond the scope of this paper, but they overwhelmingly outperform serologic tests (Goud et al., 2022; Saleh et al., 2017). Alpha-defensin is the most exhaustively studied option, and it has even been attributed a place in the most recent PJI definitions (McNally et al., 2021; Shohat et al., 2019). However, its commercially available test kit has been developed using the old Musculoskeletal Infection Society (MSIS) definition as the gold standard, and hence appears to lack sensitivity when evaluated using more sensitive definitions of infection (Chen et al., 2019; Renz et al., 2018). In addition, it is expensive and has not been shown to offer a significant advantage over traditional synovial fluid analysis (Ivy et al., 2021). The same holds for most other commercially available point-of-care tests.

For the past few years, we have been routinely measuring simple and inexpensive synovial fluid biomarkers, such as CRP, ADA, and A2M, and we have shown them to be of added value (Sousa et al., 2017). These are molecules that we can easily ask our technicians to add to the results in our laboratory, and it costs us about the same as performing a differential leukocyte count.

C-reactive protein is an acute phase reactant, produced in the liver, that has long been used as a serum biomarker in the diagnosis of PJI. Several studies have shown it to be present in higher concentrations in the synovial fluid of infected joints when compared to aseptic failures (Sousa et al., 2017; De Vecchi et al., 2018; Omar et al., 2015; Tetreault et al., 2014; Wang et al., 2021; Plate at al., 2019). Although its role as a single-standing diagnostic tool is not clear, it has been extensively studied as an adjunct, especially with alpha-defensin, with proven benefits (Deirmengian et al., 2014; Stone et al., 2018; Ettinger et al., 2020). The present study confirms our previous findings that synovial C-reactive protein may be a useful adjunct to PJI diagnosis. Validating it with the new EBJIS PJI definition, we found an optimal cut-off of 2.7 mg L ${}^{-1}$ , with a value over 8 mg L ${}^{-1}$ associated with high specificity and PPV. These thresholds are in line with some previously suggested values (De Vecchi et al., 2018; Omar et al., 2015; Tetreault et al., 2014).

Adenosine is a purine nucleoside with anti-inflammatory and tissue protective properties, and it has been proposed that the measurement of the levels of ADA in different tissues, especially in serous body fluids, may help to identify activation of the immune system (Kumar and Sharma, 2009). Zamani et al. (2012) showed that synovial fluid ADA could help distinguish inflammatory from non-inflammatory arthritic conditions and seemed to be a useful marker in differentiating septic from rheumatoid and crystal-induced arthritis. To the best of our knowledge, ADA is not a commonly used biomarker for the diagnosis of PJI. Nevertheless, using the EBJIS definition we found an helpful cutoff of 60 U L ${}^{-1}$ in line with our previous findings (Sousa et al., 2017).

A2M inhibits the excess of proteinases released by neutrophils or pathogens during tissue injury (Sousa et al., 2017). We also tested the role of A2M and found an optimal cut-off of 420 mg L ${}^{-1}$ , with an accuracy value of 82.1 %. Jacovides (2011) found an accuracy value of 89.5 % for a threshold of 0.262 in diagnosing infected PJI, a cutoff value much lower than ours. Its value significantly increases between groups and raises the specificity when combined with leucocyte count and PMN %. It demonstrated to be the worst biomarker with the lower diagnostic power in PJI diagnosis in our study.

Most importantly, this study highlights the importance and added value of combined interpretation of several different synovial fluid parameters. This is especially useful when adopting the EBJIS PJI definition philosophy to make preoperative decisions. Lower-end cutoffs were shown to reliably rule out infection, especially if more than one of the parameters are negative (low total leukocyte count or proportion of PMN and synovial fluid CRP or ADA). On the other hand, higher-end cutoffs manifested very high PPV. However, in between these values there is a grey area of interpretation. The results of the current study show that if at least two parameters are above the lower cutoff (i.e. leukocyte count >1470 , PMN >62.5% , synovial CRP >2.7 mg L ${}^{-1}$ , ADA >60 U L ${}^{-1}$ ), the PPV for PJI actually being present is very high. When three out of four tests were positive, specificity and PPV were as high as 100 %. These results emphasise the utility of simple and inexpensive biomarkers, especially in inconclusive results, in conducting our decisions.

The findings of the present study must be viewed in the context of several limitations. First, because there is no gold standard diagnosis of PJI, any diagnostic accuracy testing will have to rely on a predetermined definition. Our choice of the EBJIS definition to validate synovial fluid results may be considered flawed because in this definition a high leukocyte count or proportion of PMN is enough to confirm infection. However, there was only one case where diagnosis was based solely on elevated leukocyte count and no other confirmatory criteria. Second, the present study used a stringent inclusion criteria. This in turn may have reduced the generalisability of our findings. However, this may also be perceived as a strength, as it eliminates biases related to confounding factors (e.g. previous antibiotic therapy, inflammatory arthritis). Third, while it may seem logical to assume that most of the patients excluded for insufficient investigation had a low clinical suspicion of infection and were thus aseptic, this may have resulted in selection bias that in turn affected the diagnostic accuracy of our estimations. Last, laboratory methods utilised to measure synovial fluid biomarkers, specifically dilution to decrease the viscosity of the synovial fluid for biochemical analysis, are also a possible source of bias.

## 6 Conclusions

While there is no gold standard test alone to diagnose PJI, we believe synovial fluid analysis, especially preoperatively, is a critical step in differentiating between infection and aseptic failure. Although many different biomarkers are already being used, differential leukocyte count is still the most accurate and widely available test. In the present study, total leukocyte count and proportion of PMN cutoffs proposed by the EBJIS definition performed well in ruling out ( <1500 cells µ L ${}^{-1}$ ) and ruling in ( >3000 cells µ L ${}^{-1}$ ) PJI. Adding simple and inexpensive biomarkers such synovial CRP or ADA and combined interpretation can be helpful in the context of inconclusive results (1500–3000 cells µ L ${}^{-1}$ ). Expensive or laborious tests are unnecessary and should be reserved for select cases where their potential benefits outweigh their limitations (Amanatullah et al., 2020).

## Data Availability

Data are available from the corresponding author after a reasonable request and after seeking permission from our institutional research department.
